# Enhancing Vascular Health and Lowering Blood Pressure in Spontaneously Hypertensive Rats through Syrah Grape (*Vitis vinifera)* Pomace: The Role of Phenolic Compounds

**DOI:** 10.3390/nu16142312

**Published:** 2024-07-18

**Authors:** Kelly C. M. da Costa, Lorrayne de S. Oliveira, Júlia C. Silva, Taynara S. Santana, Raiany A. de Freitas, Alecsander F. M. Bressan, Sérgio Gómez-Alonso, José Pérez-Navarro, Paula B. Pertuzatti, Fernanda R. Giachini

**Affiliations:** 1 Institute of Biological and Health Sciences, Federal University of Mato Grosso, Barra do Garças 78060-900, Brazil; 2 Institute of Exact and Earth Sciences, Federal University of Mato Grosso, Barra do Garças 78060-900, Brazil; 3 Regional Institute of Applied Scientific Research, University of Castilla-La Mancha, 13071 Ciudad Real, Spainjose.pnavarro@uclm.es (J.P.-N.); 4 Institute of Biological Sciences, Federal University of Goias, Goiania 74690-900, Brazil

**Keywords:** winery waste, flavonols, hydroxycinnamic acids, grape pomace, lees, hypertension

## Abstract

Background: The beneficial properties of wine by-products include actions that help prevent and treat cardiovascular conditions such as hypertension, primarily due to their antioxidant effects. Novel pharmacotherapies are being developed to treat arterial hypertension, including investigations into natural products exhibiting biological activity, necessitating rigorous evaluation of their efficacy and safety. This study aimed to identify and quantify phenolic compounds in Syrah (*Vitis vinifera*) grapes grown in the Brazilian Cerrado and their presence in winemaking by-products. It also examined the effects of grape pomace on blood pressure. Methods: Fresh grapes, pomace, and lees, were subjected to spectrophotometric determination of total phenolic compounds, followed by identification and quantification using HPLC-DAD-ESI-MSn. Normotensive male rats (Wistar) and spontaneously hypertensive rats (SHR) received grape pomace-enriched (150 or 300 mg/kg/day, 14 days) or standard chow. Indirect arterial pressure was assessed, while vascular reactivity was evaluated in mesenteric resistance arteries. Results: Pomace samples exhibited higher total phenolic compound concentrations than grapes or lees. Seven derivatives of hydroxycinnamic acids and twenty-one flavonols were identified. Quercetin-3-glucoside and ethyl caffeate were the most abundant phenolic compounds. Grape pomace-enriched chow demonstrated a dose-dependent hypotensive effect in rats. Conclusion: the abundance of flavonols and hydroxycinnamic acids, combined with their hypotensive effects, underscores the therapeutic potential of fine wine-making by-products produced in the Brazilian Cerrado.

## 1. Introduction

The agroindustry stands as a basis of the Brazilian economy, significantly contributing to global food supplies. In parallel, the waste generated by this activity imposes environmental challenges, and therefore, aligning agroindustry production with the environmental agendas requires great effort [1,2]. In 2019, global grape production accounted for 8% of the total fruit harvest and Brazil produced 1.4 million tons of grapes, cultivated in almost 74 thousand hectares. Brazilian grape cultivation primarily targets table wine production, generating a considerable amount of wine by-products, generally treated as waste [3]. Nevertheless, the production of fine wine from *Vitis vinifera* grapes has gained power, especially in regions that are not traditional wine producers, including the Brazilian Cerrado.

Besides the fact that wine by-products are generally discarded, it is known that several bioactive compounds are found in them. Compounds, such as flavonols and stilbenes, are frequently identified in wine by-products and some of them are related to antioxidant capacity, as evidenced in previous studies [4,5]. The beneficial properties of wine by-products include desirable actions to prevent and treat cardiovascular conditions, such as hypertension. These actions include its antioxidant effect, anti-inflammatory effect [6], free radical scavenging abilities [7], action as an anti-proliferative agent, and ability to evoke vasodilation [8].

Systemic arterial hypertension (SAH), a major risk factor for cardiovascular diseases, accounts for several deleterious outcomes in a substantial portion of the global population. Approximately one-third of hypertensive patients do not receive adequate pharmacological treatment [9]; moreover, a significant proportion of hypertensive patients are resistant to conventional pharmacotherapy, eliciting the search for novel treatments. In this context, the investigation into natural products exhibiting biological activity has attracted considerable attention, necessitating rigorous evaluation of their efficacy and safety [10,11,12].

Despite the composition of bioactive compounds in some wine by-products still being available for some grape varieties, the composition of these bioactive products may vary due to several parameters, including grape variety, geographic location, and vinification technique, among others. Indeed, the phytochemical profile from wine-making by-products produced in the Brazilian Cerrado is not available and should be encouraged, to clarify the compositional characteristics and potential use of these residues.

Hence, this study aimed to identify and quantify phenolic compounds in *Vitis vinifera* grapes, specifically the Syrah variety cultivated in the Brazilian Cerrado, and wine-making by-products such as grape pomace and lees. Additionally, this study evaluated the effects of grape pomace treatment on blood pressure and vascular function in hypertensive rats.

## 2. Material and Methods

### 2.1. Sample Collection and Storage

Grape samples (2 kg) of Vitis vinifera grapes (Syrah) and its wine-making by-products—pomace (5 kg) and lees (5 kg)—were collected during the October 2022 harvest from a winery situated in the municipality of Cocalzinho de Goiás, Goiás, Brazil. The winery is positioned at coordinates 15°47′41′′ South and 48°46′41′′ West, with an altitude of 1085 m. The region exhibits a tropical climate with maximum temperatures of 31 °C and minimum temperatures of 15 °C. Upon collection, the samples were placed in plastic bags and stored at −20 °C until further analysis.

### 2.2. Ultrasound-Assisted Extraction of Samples

Two grams of grape samples and crushed grape pomace, along with lees samples, were individually weighed in triplicate. Subsequently, 25 mL of the extracting solution [methanol, water, and formic acid (50:48.5:1.5 *v*/*v*/*v*)] was added to each sample. The samples were then subjected to ultrasound-assisted extraction (15 min, 60 Hz) in an ultrasonic bath, followed by vacuum pump filtration. The resulting solution underwent a second extraction process, bringing the final volume to 50 mL before subsequent analyses were performed.

### 2.3. Bioactive Compounds

#### 2.3.1. Determination of Total Phenolic Compounds

The concentration of total phenolic compounds (TPCs) was determined using the Folin–Ciocalteu reagent protocol, following the method adapted from Singleton and collaborators [13]. The analysis was conducted in triplicate, with a dilution factor of 1:25 for the analyzed extracts. After the reaction period (120 min), absorbance was measured (760 nm). The results were calculated using a gallic acid (0.01–0.08 mg/mL) standard curve equation and expressed as milligrams of gallic acid (GAE) per 100 g of dry sample.

#### 2.3.2. Determination of Total Monomeric Anthocyanins

The quantification of monomeric anthocyanins (ACY) was performed using the differential pH method, outlined by Giust and Wrolstad [14]. The extracts were fully incorporated into two distinct solutions: a potassium chloride buffer solution (0.025 mol/L, pH 1) and a sodium acetate solution (0.4 mol/L, pH 4.5). The extracts were diluted (1:4), and the absorbance was accessed (520 and 700 nm). The calculation was based on the molecular weight of malvidin-3-glucoside (M3G; 494 g/mol) and its molar absorptivity [(ε) = 36,400]. Results are reported as total milligrams of M3G per 100 g of sample.

#### 2.3.3. Determination of Condensed Tannins

Condensed tannins were determined following the method adapted from Sarneckis and collaborators [15]. This colorimetric method involved adding a methylcellulose solution (0.04%) to the sample extract in a 1:1 volume ratio, which was then diluted in a 1:10 ratio with the extracting solution. Subsequently, saturated ammonium sulfate solution (2 mL) and distilled water (6 mL) were added to the mixture. The solution was then centrifuged for 5 min at 4000 rpm and the absorbance was measured at 280 nm using a spectrophotometer. To determine the content of condensed tannins, a standard curve of epicatechin (10–250 mg/L) was constructed, and the results are expressed in milligrams of epicatechin per 100 g of sample.

#### 2.3.4. Determination of Total Carotenoids

Total carotenoids were determined using Rodriguez-Amaya’s method [16]. This involved incorporating cold acetone (1:4–1:6 *w*/*v*) into the sample and performing exhaustive extraction through filtration with a vacuum pump. This extraction process was repeated until the color of the sample was completely depleted. Subsequently, partitioning was carried out using a separation funnel containing petroleum ether (25 mL) to form the upper phase, and acetone forming the lower phase. Distilled water was added to the upper nozzle of the funnel repeatedly to wash the sample until the acetone was completely removed, leaving only the upper phase containing the carotenoids. The absorbance was measured at a wavelength of 450 nm. Results are expressed as micrograms of β-carotene per gram of sample, using the molar extinction coefficient of β-carotene in petroleum ether (2592 m^2^/mol).

### 2.4. Phytochemical Analysis 

#### 2.4.1. Extraction of Non-Anthocyanin Phenolic Compounds for Analysis by HPLC-DAD-ESI-MSn

In natura grape samples and pomace, both subjected to freeze-drying, and lees, kiln-dried (obtained from an oven at 40 °C), were utilized for extraction. These samples underwent extraction with a solution comprising methanol (1:10 *w*/*v*), water, and formic acid (50:48.5:1.5 *v*/*v*/*v*), employing an ultrasonic bath for 2 min, followed by centrifugation at 5000 rpm (5 °C) for 10 min. The resulting extracts were filtered and stored at −18 °C until further use.

To obtain anthocyanin-free extracts, Solid Phase Extraction (SPE) cartridges (Bond Elut Plexa PCX, Agilent Technologies, Santa Clara, CA, USA) packed with 500 mg of cation-exchange polymeric adsorbent resin, were employed. Elution was performed using 6 mL of 96% ethanol to collect the non-anthocyanin phenolic fraction [17]. Each extract (3 mL sample) was subsequently dried in a rotary evaporator (35 °C), reconstituted in aqueous methanol solution (1:5; *v*/*v*), filtered through a polyester membrane (0.20 μm), and directly injected into the HPLC-DAD-ESI-MSn system for phenolic compound determination. The extracts were pipetted in 0.5 mL aliquots and eluted in 0.5 mL HCl (0.1 N) solution.

#### 2.4.2. Identification and Quantification of Non-Anthocyanin Phenolic Compounds by HPLC–DAD-ESI–MSn

Phenolic compound identification and quantification were conducted using the Agilent Series 1100 HPLC system equipped with a DAD diode detector and an electrospray ionization mass spectrometry system (ESI-MSn) LC/MSD Trap VL, coupled to an Agilent Chem Station data processing station. The solvent system comprised water/acetonitrile/formic acid (solvent A: 88.5:3:8.5 *v*/*v*/*v*; solvent B: 41.5:50:8.5 *v*/*v*/*v*) at a flow rate of 0.16 mL/min. A linear gradient for solvent B was employed as follows: 4% at 0 min, 30% at 15 min, 35% at 20 min, 35% at 30 min, 100% at 35 min, 100% at 40 min, and 4% at 45 min. Phenolic compounds were analyzed using negative ionization mode ESI-MSn, with parameters including the dry gas (N2, 8 L/min), drying temperature (325 °C), nebulizer pressure (N, 50 psi), capillary voltage (4500 V), skimmer 1 (15 V), and skimmer 2 (6 V). The scanning range was set at 100–1200 *m*/*z*. Samples were injected (20 μL) into a Zorbax Eclipse XDB-C18 reversed-phase column (2.1 × 150 mm; 3.5 μm particle, Agilent), maintained at 40 °C. Flavonols and hydroxycinnamic acid (HCAD) derivatives were identified based on their UV-Vis and MS/MS spectral data, compared to authentic standards. Flavonol quantification utilized DAD chromatograms extracted at 360 nm, expressed as quercetin-3-glucoside (Q-3-glc) equivalents. HCAD was quantified using chromatograms at 320 nm and expressed as caftaric acid equivalents.

### 2.5. Methodology for Biological Tests

All experimental procedures were conducted following approval on 5 May 2023, by the Animal Ethics Committee of the Federal University of Mato Grosso (CUA-UFMT), under protocol number 23108.010699/2023-18. The experiments were conducted at the Vascular Biology and Histopathology Laboratory and the Food Analysis Laboratory, both located at the Federal University of Mato Grosso.

#### 2.5.1. Production and Characterization of Experimental Chow

Standard chow pellets (Nuvilab^®^—QUIMITIA SA, Colombo, Brazil) were enriched with grape pomace, prepared at two different concentrations (0.75% and 1.5%), designated as Grape Pomace 1 (GP1) and Grape Pomace 2 (GP2), respectively. Balanced chow composition is available in the Supplemental Material. The grape pomace was thawed, weighed, crushed, and incorporated into crushed standard commercial laboratory chow, ensuring uniform distribution of grape pomace within the chow matrix. Subsequently, the homogeneous mixtures were moistened and manually shaped into pellets, resembling conventional chow. These pellets were dried (45 °C) in an oven with air circulation (3 h). The grape pomace-enriched chow was produced weekly and stored (5 °C) until use.

#### 2.5.2. Determination of Chow Total Phenolic Content

The characterization used 5 g of each sample, in triplicate, which were submitted to extraction according to Section 2.2. Total phenolic compound (TPC) concentration determination followed the protocol described in Section 2.3.1 (1:1; dilution factor).

#### 2.5.3. Animals

Male normotensive Wistar and hypertensive rats [spontaneously hypertensive rats (SHR)], weighing between 250 and 300 g (8–10 weeks old) were used. All rats were housed at the Animal Maintenance and Experimentation Center (23108.010699/2023-18 CEMAE-UFMT/CUA), under controlled conditions of temperature (20 ± 2 °C) and circadian rhythm (12-hour light–dark cycle), with free access to water and chow. Throughout the 14-day treatment period, rats from different experimental groups were housed individually.

Normotensive and hypertensive rats were randomly assigned to homogeneous groups, based on their blood pressure and type of diet, resulting in the following groups:

Normotensive Control (NC, n = 6); Hypertensive Control (HC, n = 6); Normotensive Grape Pomace 1 (NGP1, n = 7); Hypertensive Grape Pomace 1 (HGP1, n = 7); Normotensive Grape Pomace 2 (NGP2, n = 7); and Hypertensive Grape Pomace 2 (HGP2, n = 7).

The daily treatment regimen for the groups ensured unrestricted access to feed consumption while also ensuring the intake of enriched feed specific to each experimental group. The treatment protocol proceeded as follows: (a) NC and HC rats received exclusively standard chow pellets, 20 g at 8 a.m., and 20 g at 6 p.m.; (b) NGP1 and HGP1 rats received up to 20 g of enriched chow pellets (0.75% grape pomace)—adjusted to 150 mg/kg/day—at 8 a.m., and 20 g of standard chow pellets at 6 p.m.; (c) NGP2 and HGP2 rats received up to 20 g of enriched chow pellets (1.5% grape pomace)—adjusted to 300 mg/kg/day—at 8 a.m., and 20 g of standard chow pellets at 6 p.m.

Before replacing the standard food in the second daily period (6 p.m.), the researchers checked whether the enriched food was consumed. Rats from NGP1, NGP2, HGP1, and HGP2 groups were treated with enriched chow adjusted weekly to their respective dosage, considering their body weights.

#### 2.5.4. Assessment of Blood Pressure Levels

Systolic blood pressure (mmHg) was assessed using a noninvasive method in awake rats, employing tail-cuff plethysmography. Rats underwent an acclimatization period before the systolic blood pressure measurement. This involved heating the animals in a box at 28 °C for 5 min and, subsequently, placing them in an acrylic cylinder with openings for the snout and tail for 5 min. After four consecutive days of adaptation, systolic blood pressure was evaluated by a pressure measurement system (tail-cuff plethysmograph V2.11 Insight). The systolic blood pressure was assessed on days 0 and 14 of the treatment with pomace grape. The final systolic blood pressure value for each animal was determined as the arithmetic mean of three consecutive measurements.

#### 2.5.5. Vascular Reactivity

After 14 days of treatment, rats were anesthetized with a mixture of 10% ketamine hydrochloride and 2% xylazine hydrochloride (60 mg/kg and 10 mg/kg, respectively, i.p.), and subsequently euthanized in a CO_2_ chamber upon confirmation of sedation. Following confirmation of animal death, a laparotomy was performed to externalize the mesentery. Mesenteric resistance arteries (3rd and 4th order) were quickly dissected, cleaned of perivascular tissue, and placed into Krebs solution (−4 °C). Arterial segments were cut into rings (4 mm in length) and mounted as ring preparations in standard organ chambers for recording isometric tension using a data acquisition system (Power Lab 8/SPAD Instruments PtyLtd., Colorado Springs, CO, USA). The segments were adjusted to maintain a passive force of 5 mN and incubated in Krebs solution, at 37 °C, continuously aerated with a mixture of CO_2_ (5%) and O_2_ (95%). Arterial integrity was assessed by stimulating the segments with KCl solution (120 mmol/L). After washing and stabilization, endothelial function was assessed by contracting the segments with Phenylephrine (PE; 1 μmol/L) followed by acetylcholine (ACh 10 μmol/L). Segments showing relaxation below 80%, in response to ACh, were excluded. Concentration–response curves were generated for contractile stimulus with FE (1 nmol/L to 100 μmol/L); for endothelium-dependent relaxation with ACh (1 nmol/L to 100 μmol/L); and for endothelium-independent relaxation with sodium nitroprusside (NPS; 0.1 nmol/L–10 μmol/L).

### 2.6. Statistical Analysis

Results are presented as mean ± standard deviation (SD) for bioactive compound analyses (n = 3) and as mean ± standard error of the mean (SEM) for biological analyses (n = 7–8). Concentration–response curves were fitted to a sigmoidal shape using non-linear regression analysis on a logarithmic scale, yielding two pharmacological parameters: maximum effect (Emax) and -log EC50 (pD2). Statistical analysis for Emax and pD2 values was performed using one-way analysis of variance (ANOVA) followed by Tukey’s post hoc test, as specified in the figure legends. A *p*-value “<0.05” was considered statistically significant.

## 3. Results

### 3.1. Spectrophotometric Analysis of Bioactive Compounds

The evaluation of total phenolic compounds, condensed tannins, total monomeric anthocyanins, and carotenoids was evaluated, and higher concentrations were found in the grape pomace samples compared to grapes; however, carotenoid concentration was similar between grape and grape pomace. Interestingly, these compounds were less concentrated in lees when compared to both grapes and grape pomace (Table 1).

### 3.2. Chromatographic Analysis of Non-Anthocyanin Phenolic Compounds

**Figure 1:**
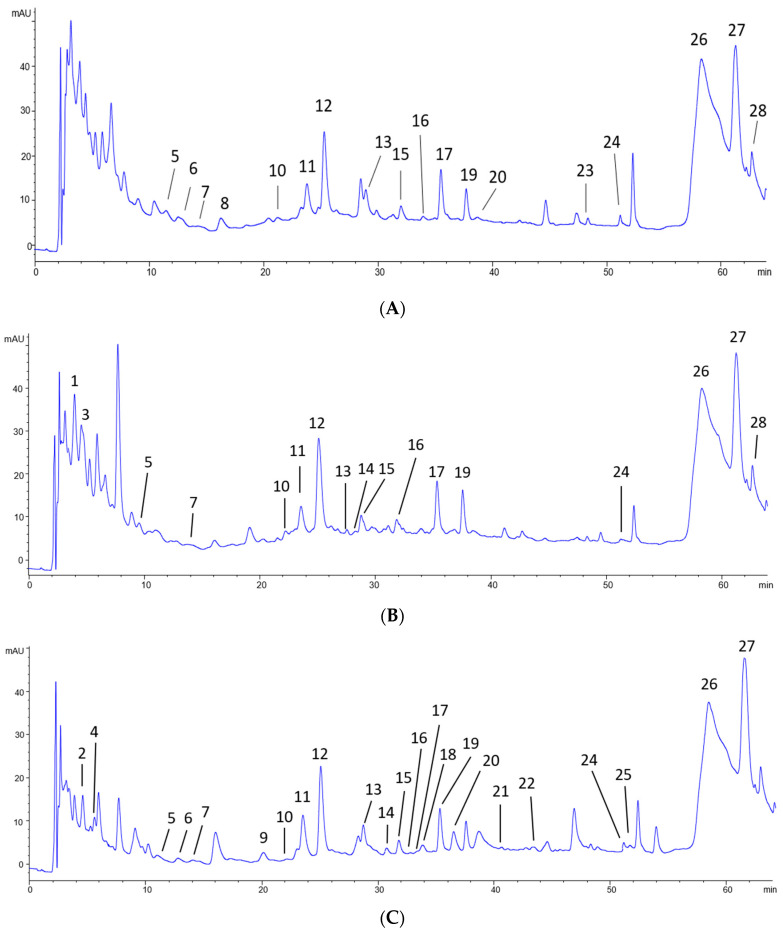
DAD chromatograms corresponding to the profile of flavonols and hydroxycinnamic acid derivatives (detected at 280 nm) of grape (**A**), grape pomace (**B**), and lees (**C**). Peaks identified are referenced in Table 2.

Total flavonols were found quantified in equivalents of (Q-3-glc mg/kg sample) in lees (111.16 ± 7.43), grape pomace (68.65 ± 12.03), and grape (60.08 ± 22.20). The flavonols identified in the chromatographic analysis are derived from six aglicones, commonly present in V. vinifera species: Kaempferol (K; *m*/*z* 285), Quercetin (Q; *m*/*z* 301), Isorhamnetin (I; *m*/*z* 315), Myricetin (M; *m*/*z* 317), Laricitrin (L; *m*/*z* 331), and Syringetin (S; *m*/*z* 345). These flavonols are predominantly found in glycosidic forms, associated predominantly with galactosides (gal) and glucosides (glc). Less commonly, these flavonols were conjugated with other glycosides, such as glucuronide (glcU), rutinoside (rut), rhamnoside (rhm), and two sugars not identified, here named hexoside (hex) and dihexoside (dihex) (Table 2 and Figure 1).

The distribution of the 21 flavonols identified in the samples was diverse, where Q-3glc, I-3-glc and S-3-gal were the most abundant flavonols found. In summary, 11 compounds were present in all samples (M-3-glcU, M-3-glc, Q-3-gal, Q-3-glcU, Q-3-glc, L-3-glc, K-3-glc, I-3gal, I-3-glc, S-3-gal, and S-3-rut): 2 compounds were present in grapes and lees but not grape pomace (M-3-gal and S-3-glc); 2 compounds were found exclusively in grapes (M-3-dihex and I-3-rut); 1 compound was found exclusively in grape pomace (L-3-gal); and 5 compounds were found exclusively in the lees (M-3-rhm, I-3-hex, Free Q, Q-3-rut, and Free I).

Regarding the HCADs, these were quantified in equivalents of caftaric acid mg/kg sample and were found in grape pomace (150.91 ± 8.18), lees (139.00 ± 1.25), and grape (132.15 ± 3.23). Ethyl caffeate and trans-caftaric acid were the HCADs with higher concentrations but seven different compounds were found in the samples including caffeic acid, coutaric acid, and fertaric acid. Among the HCADs identified, two compounds were present in all samples (ethyl caffeate and trans-caftaric acid); one compound was present in grapes and grape pomace (cis-caftaric acid); two compounds were found exclusively in the grape pomace (trans-caffeic acid and cis-caffeic acid); and two compounds were found exclusively in the lees (cis-coutaric acid and trans-fertaric acid) (Table 2).

### 3.3. Grape Pomace-Enriched Chow and Biological Assays

**Figure 2:**
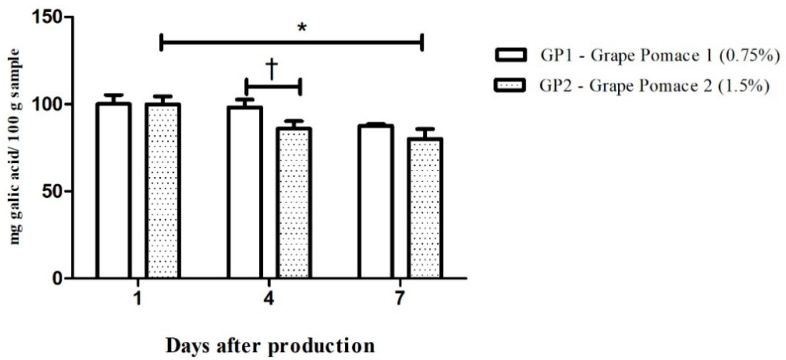
Evaluation of the phenolic compound content in chow enriched with 0.75% (white bars) or 1.5% (dot bars) of grape pomace. Values are expressed as means ± SD (n = 5). Comparisons were conducted using one-way analysis of variance (ANOVA), followed by Tukey’s post hoc test. * *p* < 0.05 vs. Grape Pomace 1 (0.75%) day 1; ^†^ *p* < 0.05 vs. Grape Pomace 2 (0.75%) day 4.

After completing the bioactive compound analyses, the enriched chow was obtained, and the total phenolic stability was assessed. It was observed that the total phenolic compound levels remained stable in the chow enriched with 0.75% grape pomace over the 7-day evaluation; however, in the chow enriched with 1.5% grape pomace, the content of phenolic compounds was reduced on the fourth day (vs. Grape I, day 4); and by the seventh day (vs. Grape II, day 1) (Figure 2).

**Figure 3:**
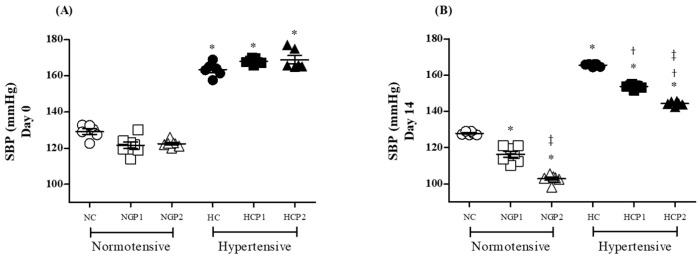
Analysis of systolic blood pressure levels (SBP) on days 0 (**A**) and 14 (**B**) of treatment in normotensive (white symbols) and hypertensive (black symbols) rats from the control group (circle), Grape Pomace 1 (square), or Grape Pomace 2 (triangles). Values are presented as means ± SD (n = 7–8). Comparisons were conducted using one-way analysis of variance (ANOVA), followed by Tukey’s post hoc test. * *p* < 0.05 vs. NC group; ^†^ *p* < 0.05 vs. HC group; ^‡^ *p* < 0.05 vs. HPG1 group.

Following stability tests for the presence of total phenolic compounds in the diets, the experimental groups were treated as previously described. On day 0, normotensive rats (NC, NGP1, and NGP2) exhibited normal blood pressure (BP) levels, while hypertensive rats (HC, HGP1, and HGP2) displayed elevated blood pressure levels compared to normotensive rats (Figure 3A), as expected.

After 14 days of treatment, the normotensive rats treated with enriched chow (NGP1 and NGP2) exhibited a dose-dependent reduction in blood pressure levels compared to the control group (NC). Similarly, hypertensive rats treated with enriched chow (HGP1 and HGP2) also displayed a dose-dependent blood pressure reduction, compared to their respective control group (HC); however, the blood pressure reduction observed after grape pomace treatment did not normalize the blood pressure levels in hypertensive groups when compared to the normotensive control group (Figure 3B).

**Figure 4:**
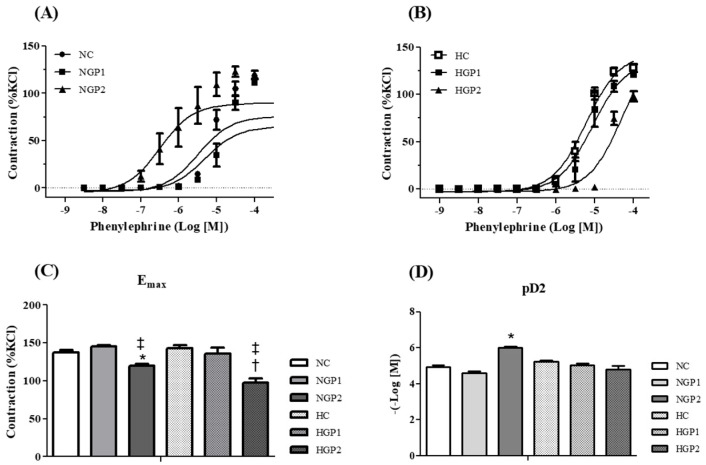
Chow enriched with pomace improves the contraction response to phenylephrine (PE) in both hypertensive rats and normotensive rats. Concentration–response curves for PE were performed in mesenteric resistance arteries from the following: (**A**) NC group (white bar), NGP1 (light gray bar), NGP2 (dark gray bar); (**B**) HC group (white bar with dots), HGP1 (light gray bar with dots), HGP2 (dark gray bar with dots). The (**C**) Emax and (**D**) pD2 values were obtained for all curves. Results are expressed as the mean ± SEM (n = 7–8). Comparisons were conducted using one-way analysis of variance (ANOVA), followed by Tukey’s post hoc test. * *p* < 0.05 vs. NC group; ^†^ *p* < 0.05 vs. HC group; ^‡^ *p* < 0.05 vs. HGP1 group.

Since blood pressure modifications are inversely related to vascular resistance, resistance arteries were used to assess vascular reactivity to contractile and relaxation stimuli. It is observed that the contractile response to PE (Figure 4A,B) was similar between the normotensive and hypertensive control groups (NC and HC). The HGP2 but not the HGP1 group was able to reduce the PE-induced Emax contractile response when compared to the HC group. In normotensive rats, a similar effect was observed, where the NGP2 group displayed a reduced PE-induced Emax contractile response, either compared to the NC or NGP1 (Figure 4C). Interestingly, a lower pD2 value was noted during the concentration–response curve to PE in the NGP2 group, compared to NC or NGP1 (Figure 4D).

**Figure 5:**
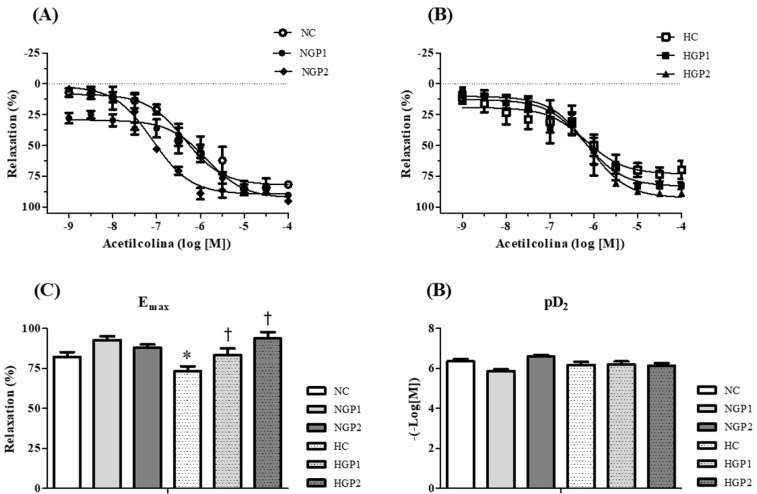
Chow enriched with grape pomace improves the endothelium-dependent relaxation response in hypertensive rats. Concentration–response curves for acetylcholine (ACh) were performed in mesenteric resistance arteries contracted with phenylephrine (PE) in the following: (**A**) NC group (white bar), NGP1 (light gray bar), NGP2 (dark gray bar); (**B**) HC group (white bar with dots), HGP1 (light gray bar with dots), HGP2 (dark gray bar with dots). The (**C**) Emax and (**D**) pD2 values were obtained for all curves. Results are expressed as the mean ± SEM (n = 7–8). ACh-induced relaxation values were calculated relative to the change in PE-evoked maximum contraction, which was taken as 100%. Comparisons were conducted using one-way analysis of variance (ANOVA), followed by Tukey’s post hoc test. * *p* < 0.05 vs. NC group; ^†^ *p* < 0.05 vs. HC group.

The endothelium-dependent relaxation, assessed by ACh-induced relaxation, showed a smaller relaxation response in the HC group compared to the NC group. The HGP1 and HGP2 groups displayed improved Emax relaxation response, in a dose-dependent feature, compared to the HC group (Figure 5A,B). In normotensive rats, no difference was observed among the groups (Figure 5C) and pD2 was similar in all groups (Figure 5D).

**Figure 6:**
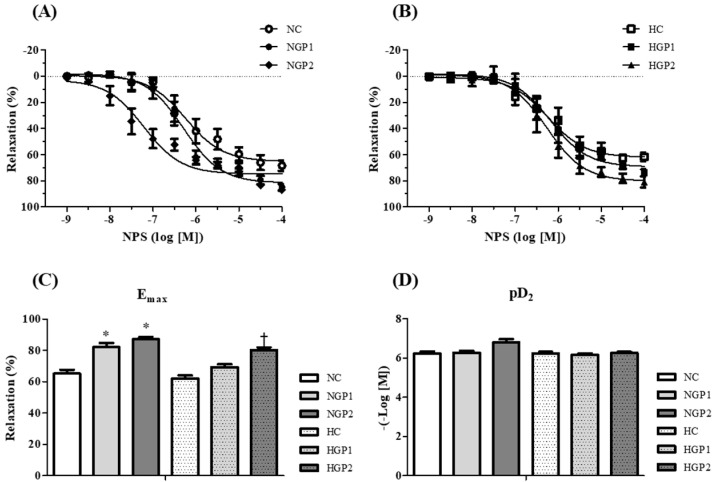
Chow enriched with grape pomace improves the endothelium-independent relaxation response in hypertensive and normotensive rats. Concentration–response curves for sodium nitroprusside (SNP) were performed in mesenteric resistance arteries contracted with phenylephrine (PE) in the following: (**A**) NC group (white bar), NGP1 (light gray bar), NGP2 (dark gray bar); (**B**) HC (white bar with dots), HGP1 (light gray bar with dots), HGP2 (dark gray bar with dots). The (**C**) Emax and (**D**) pD2 values were obtained for all curves. Results are expressed as the mean ± SEM (n = 7–8). SNP-induced relaxation values were calculated relative to the change in PE-evoked maximum contraction, which was taken as 100%. Comparisons were conducted using one-way analysis of variance (ANOVA), followed by Tukey’s post hoc test. * *p* < 0.05 vs. NC group; ^†^ *p* < 0.05 vs. HC group.

The relaxation response evoked by NPS, related to the endothelium-independent relaxation response, was similar between NC and HC (Figure 6A,B). The HGP2 group increased Emax relaxation induced by NPS when compared to the HC. NGP1 and NGP2 increased Emax relaxation induced by NPS when compared to the NC group (Figure 6C). pD2 was similar among the groups evaluated (Figure 6D).

## 4. Discussion

In the analysis of total phenolic compounds, it was observed that grape pomace exhibited a concentration twice as high as that of grapes, which aligns with previous studies highlighting grape pomace from wine-making as a significant source of total phenolic compounds. Even after the pressing and maceration processes, a considerable amount of these biomolecules remained in the grape pomace, as they are not entirely transferred during wine-making, thus contributing to the generation of this biomolecule-enriched type of residue. Additionally, factors such as the genotypic characteristics of the grape, cultivation methods, and fermentation processes influence the total phenolic content found in grape pomace [18,19,20]. Furthermore, our findings demonstrate that grape pomace derived from the vinification of Vitis vinifera grapes, specifically from the Syrah variety, exhibited a higher concentration of tannins, anthocyanins, and carotenoids compared to lees or grape samples. The grape pomace utilized in this study comprised seeds, skin, and grape stems. Previous reports indicate that condensed tannins can constitute up to 52% of the weight of the residue, primarily due to the prevalence of seeds in skin, which represent the richest source of tannins within the grape [18]. Kammerer and collaborators reported that the total anthocyanins present in Cabernet Mitos grape pomace can reach up to 38% of the total amount found, underscoring pomace as an important source of these compounds. This is because the transfer of anthocyanins to wine is less than 40% due to the solid–liquid partition coefficients of individual compounds, which significantly influence this process [21,22]. Carotenoids found in skins and seeds contribute up to 11% of the grape’s weight, making them more concentrated in the pomace [18].

Given these findings, we employed HPLC-DAD-ESI-MSn, a more specific technique for the identification and quantification of these compounds. This technique offers greater sensitivity and selectivity for identifying phenolic compounds compared to colorimetric assays [23].

From the results obtained, M-3-dihex compounds (7.26%) and I-3-rut (1.60%) were identified exclusively in grapes, while grape pomace exhibited the presence of L-3-gal (2.88%), found exclusively in this sample. The most abundant flavonols, Q-3-glc and I-3-glc, were present in all three samples in greater quantities, with pomace registering the highest quantification levels (35.99% and 15.68%), respectively.

Quercetin-derived flavonols were predominant in the samples, consistent with findings from studies analyzing residues such as skin, pomace, and lees from various grape species [1,24,25,26,27]. The lees exhibited unique compounds in their profile—such as M-3-rhm (4.27%), I-3-hex (5.01%), and Q-3-rut (1.23%)—not identified in the grape or grape pomace. This characteristic profile can be attributed to the fermentation process carried out by yeasts during vinification, as they can break chemical structures and modify molecules, particularly in the lees [28,29,30]. Free aglycones Q (0.87%) and I (5.73%) were also found in the lees, a common result for this type of residue, possibly due to the hydrolysis of the respective flavonol 3-glucoside during winemaking. Flavanol aglycones have low solubility, and therefore, in hydroalcoholic solutions such as wine, their partition coefficient does not facilitate the complete transference of these compounds to the wine, leading to their accumulation in the lees [17,31,32]. The lees exhibited a greater variety of individual flavonols, with 18 types found, while 13 types were found in the grape pomace, and 15 in the grape. These results demonstrate that both grape pomace and lees display a higher phenolic composition than grapes. When compared to each other, the lees present a greater variety of flavonols than the grape pomace; however, when quantified, the pomace presents a higher molar concentration for six types of flavonols present in both samples. For this reason, we selected grape pomace to conduct the biological studies, as discussed below.

The quantification of HCADs revealed that both residues present a greater variety and quantity in their samples compared to grapes, for example, the presence of trans-caffeic acid (5%) and cis-caffeic acid (4.59%) is found only in grape pomace, and cis-coutaric acid (2.71%) and trans-fertaric acid (3.25%) is present only in the lees, a profile similar to other studies [28,33,34]. The most abundant HCAD was ethyl caffeate, found in greatest quantities in grapes, followed by lees and grape pomace.

Grape pomace exhibited a greater variety in components (flavonols and HCAD) than lees and contributed the highest proportion to total equivalents in mg/kg. These identified biomolecules constitute an important source of secondary metabolites produced in response to injuries suffered by the plant and characterize a unique identity of each species that needs to be explored and known for various purposes, including nutritional and pharmacological [35].

This variety of components, which are different in terms of quantity or composition related to other studies, can be explained by several factors associated with the biosynthesis of flavonoids in grapes. Genetics, and external factors such as environmental conditions and management (temperature, light, type of soil, water, nutritional status of the soil, climate of the growing region, winemaking practices, yield, and the steps used to produce wine) influence the concentration profile of flavonoids in grapes, as well as in their residues [35,36,37]. The climatic conditions and soil type of the Brazilian Cerrado stand out, being drastically different from regions where grape cultivation is traditional in Brazil, including the south and southeast regions. The climate of this biome is seasonal tropical, with a well-defined rainy and dry season. The soil in general is acidic and poor in nutrients [38].

Once we verified the richness of the total phenolic compounds in the grape pomace evaluated, two types of chow enriched with this by-product were created. Here, it is important to acknowledge a limitation regarding the chow enriched by grape pomace, due to the use of a non-purified diet, as it introduces variability to the study due to inconsistent chow ingredients across groups [39].

An important step was to determine the stability of the chow, proving to be viable up to the first six days after preparation; however, on the seventh day after preparation, there was a loss of phenolic composition present in the chow enriched with GP2, whereas chow produced with GP1 remained stable. Thus, although several factors can contribute to the loss of phenolic composition in foods, such as heat, moisture, and light, the enriched chow used in this study proved to be suitable for treating animals, since production was carried out weekly [40,41].

In fact, the viability of the presence of phenolic compounds in the chow coincided with an improvement in the blood pressure levels of the experimental animals. The analysis of the animals’ blood pressure levels showed that the grape pomace introduced into the chow was capable of reducing, in a dose-dependent manner, the blood pressure for both normotensive and hypertensive rats. A previous study demonstrated that the treatment of SHR rats with seedless skin—from wine-making using *V. vinifera* grapes, the Tempranillo variety—reduced systolic blood pressure by approximately 11% after 4 weeks when using the skin powder dissolved in water at a dose of 300 mg/kg/day [42]. However, previous studies did not investigate the action of supplementation in normotensive rats, or whether this treatment impacted vascular function.

Other wine-making by-products, such as lees, have also demonstrated hypotensive effects in previous studies, where wine lees powder resulted in a dose-dependent reduction in blood pressure in SHR rats, with a maximum pressor effect after 6 h of oral administration, returning to initial levels after 48 h [43]; however, these authors did not evaluate the effect of chronic use of lees, and the effect of this supplementation was not able to reduce the blood pressure of normotensive rats, as described by the authors, contradicting the results presented here.

In this sense, the present study innovates by showing that not only SBP but also vascular reactivity was improved due to the incorporation of grape pomace into the diet, both in hypertensive and normotensive rats. An important characteristic observed in arterial hypertension is the reduction in endothelium-dependent relaxation [44], confirmed in this study. Higher doses of grape pomace resulted in a reduction in the Phe-induced contractile response. Interestingly, treatment with enriched chow was able to restore endothelium-dependent relaxation, in a dose-dependent manner, in the hypertensive group. Another aspect observed was that endothelium-independent relaxation was improved in the arteries from normotensive rats, in all groups treated with enriched chow, while in hypertensive rats this effect was only observed at the highest dose (HGP2).

During hypertension, both hypercontractility and endothelial dysfunction culminate in mechanisms that promote increased vascular resistance, and consequently, blood pressure [45,46,47]. Taken together, our data demonstrate that the enriched diet contributes to a reduction in vascular resistance, both for normotensive and hypertensive rats, even though it triggers different molecular mechanisms.

The hypotensive mechanism observed in this study was dose-dependent and correlated with the large phenolic composition present in the grape pomace incorporated into the chow. Among the bioactive compounds, we know that flavonols, tannins, anthocyanins, carotenoids, and phenolic acids are capable of promoting different antihypertensive activities, including antioxidant activity, improvement of endothelial function, increased availability of nitric oxide (NO), and inhibition of the renin–angiotensin system, among others [42,48,49,50].

Another study demonstrated that the intake of compounds rich in polyphenols was able to reduce inflammation and promote remodeling of cardiac tissue, helping to prevent fibrosis and oxidative stress in aged animal groups [51]. Likewise, the introduction of Malbec grape pomace, in powder form, into the diet of Wistar rats fed a fructose and high-fat diet resulted in a notable reduction in the symptoms of cardiovascular diseases by stimulating the production of nitric oxide by the vascular endothelium. Similarly, a mixture of Cabernet Sauvignon, Syrah, and Marselan grape pomace also showed a cardiovascular protective effect in normotensive rats in the intermediate stage of adulthood [52,53]. Balea and collaborators discovered that the grape pomace Fetească neagră, a variety native to Romania, presented cardioprotective effects against myocardial ischemia caused by isoprenaline—via the generation of oxidative stress—with high levels of antioxidants being evidenced in the pomace of this grape alongside anthocyanin groups, proanthocyanidins, flavan-3-ol monomers, and stilbenes [54]. In this way, it is corroborated by evidence that these phenolic compounds present in wine residues can be presented as an alternative source for treating hypertension and cardiovascular dysfunctions.

It should also be emphasized that the predominant flavonols in the grape pomace samples of this study, such as quercetin 3-glucoside (Q-3-glc) and isorhamnetin 3-glucoside (I-3-glc), are mentioned in other studies that evaluated the ingestion of these compounds in isolation (capsules) demonstrating their direct relationship with the reduction in hypertension in hypertensive animal models, through possible mechanisms ranging from antioxidant action, interference with the renin–angiotensin–aldosterone system, or improvement in vascular function in an endothelium-dependent or -independent manner [55,56,57,58]. Another compound analyzed was ethyl caffeate, derived from HCAD, present in important concentrations in all samples. The individual vasodilatory activity of ethyl caffeate and its potential effect as an angiotensin-converting enzyme inhibitor, in vitro, may suggest a mechanism for this compound to reduce hypertension [59,60].

Given the above, we concluded that grape pomace from the winery using grapes grown in the Brazilian Cerrado has a significant number of bioactive compounds that exhibit antihypertensive and vascular protection activity. Currently, these wastes are not managed in an environmentally sustainable way, and we hope that studies like this can provide economic exploration, enabling their use in the production of medicines and other products of health interest.

## 5. Conclusions

This study highlights the significant presence of phenolic compounds, including flavonols and hydroxycinnamic acid derivatives, in grapes, grape pomace, and lees obtained from the vinification of V. vinifera grapes cultivated in the Brazilian Cerrado. Importantly, incorporating grape pomace into the daily diet of experimental animals led to a reduction in blood pressure levels in both hypertensive and normotensive rats, along with enhancements in vascular resistance. These findings spark interest in investigating how the by-products of winemaking in the Brazilian Cerrado could be used for improving cardiovascular health.

## Figures and Tables

**Table 1:** Assessment of total phenolic compounds, condensed tannins, anthocyanins, and carotenoids in samples of Syrah grapes, grape pomace, and lees.

Analysis	Grape	Grape Pomace	Lees
**Total phenolic compounds** (mg GAE/100 g sample)	350.3 ± 8.3	895.5 ± 15.8 *	102.43 ± 6.1
**Condensed tannins** (mg epicatechin/100 g sample)	1098.3 ± 48.1	2211.6 ± 207.3 *	295.60 ± 29.1 *^,†^
**Total monomeric anthocyanins** (mg M-3-glc/100 g sample)	20.84 ± 0.15	25.68 ± 1.6 *	13.47 ± 0.36 *^,†^
**Total carotenoids** (μg β-carotene/g sample)	747.33 ± 12.7	782.45 ± 55	616.30 ± 21.3 *^,†^

Values are presented as means ± SD (n = 3). Statistical comparisons were conducted using one-way analysis of variance (ANOVA), followed by Tukey’s post hoc test. * *p* < 0.05 vs. grapes; ^†^ *p* < 0.05 vs. grape pomace. Abbreviations: *GAE*, galic acid; M-3-glc, Myricetin-3-glucoside.

**Table 2:** Identification (chromatographic retention times and MSn data obtained in negative mode) and quantification of phenolic compounds found in freeze-dried samples of *Vitis vinifera* grapes, Syrah variety, and its winemaking by-products, grape, pomace, and lees, from the 2022 harvest.

Nº	Phenolics Compounds	R_t_ (min)	Molecular Ion; Product Ions (*m*/*z*) ^a^	Grape (molar%)	Grape Pomace (molar%)	Lees (molar%)
	**Flavonols**					
	Total (mg/kg sample) ^b^			60.08 ± 22.20	68.65 ± 12.03	111.16 ± 7.43 *^,†^
5	M-3-glcU	14.1	493; 317	1.40 ± 0.44	1.00 ± 0.36	1.34 ± 0.09
6	M-3-gal	14.5	479; 317	5.38 ± 0.22	ND	0.44 ± 0.04 *
7	M-3-glc	16.1	479.2; 317	3.01 ± 0.53	2.46 ± 0.99	14.93 ± 0.55 *^,†^
8	M-3-dihex	16.2	479.2; 317	7.26 ± 0.85	ND	ND
9	M-3-rhm	20	463; 317	ND	ND	4.27 ± 0.76
10	Q-3-gal	23	463.2; 301	1.73 ± 0.50	1.83 ± 0.42	0.80 ± 0.07 *
11	Q-3-glcU	23.7	477.3; 301	9.30 ± 0.50	11.24 ± 2.37	9.18 ± 0.21
12	Q-3-glc	25.3	463.3; 301	31.20 ± 1.62	35.99 ± 1.92 *	22.29 ± 0.92 *^,†^
13	L-3-glc	28.7	493; 331	4.04 ± 0.51	5.94 ± 0.65 *	4.02 ± 0.16 ^b^
14	L-3-gal	29.9	493; 331	ND	2.88 ± 0.61	ND
15	K-3-glc	31.8	447; 285	5.29 ± 0.71	5.89 ± 1.68	3.05 ± 0.16 ^†^
16	I-3-gal	33.8	477.4; 315	1.28 ± 0.34	1.67 ± 0.52	1.76 ± 0.17
17	I-3-glc	35.4	477.3; 315	14.23 ± 1.16	15.68 ± 1.44	8.08 ± 0.26 *^,†^
18	I-3-hex	36.2	477.2; 315	ND	ND	5.01 ± 0.54
19	S-3-gal	37.6	507.3; 345	10.07 ± 0.49	14.40 ± 0.88 *	6.09 ± 0.35 *^,†^
20	S-3-glc	38.6	623; 344	1.54 ± 0.12	ND	9.30 ± 0.93 *
21	Free Q	39.3	301; 301	ND	ND	0.87 ± 0.15
22	Q-3-rut	43.4	609; 301	ND	ND	1.23 ± 0.06
23	I-3-rut	48.3	623; 315	1.60 ± 0.71	ND	ND
24	S-3-rut	51.3	623; 345	2.68 ± 0.18	1.02 ± 0.18 *	1.57 ± 0.16 *^,†^
25	Free I	51.7	315;	ND	ND	5.78 ± 0.60
	**Hydroxynamic acid derivatives**					
	Total (mg/kg sample) ^c^			132.15 ± 3.23	150.91 ± 8.18 *	139.00 ± 1.25
1	Trans-caffeic acid	3.9	341; 179; 161; 135	ND	5.0 ± 1.04	ND
2	Cis-coutaric acid	4.4	295; 149	ND	ND	2.71 ± 0.11
3	Cis-caffeic acid	4.5	341; 179; 135	ND	4.59 ± 1.44	ND
4	Trans-fertaric acid	5.9	325; 193	ND	ND	3.25 ± 0.19
26	Ethyl caffeate	59.6	207; 179	77.21 ± 1.91	68.93 ± 0.78 *	70.97 ± 0.12 *
27	Trans-caftaric acid	60.7	311; 149	22.44 ± 1.82	21.09 ± 1.60	23.08 ±0.21
28	Cis-caftaric acid	62.1	311; 179	0.34 ± 0.10	0.39 ± 0.03	ND

Values are expressed as means ± SD (n = 3). Total concentrations of each type of phenolic compound are reported in milligrams of representative standard equivalent per kilogram of dry weight sample. The contribution of each individual compound within a type of phenolic compound to the total concentration is expressed in molar percentages. Comparisons were conducted using one-way analysis of variance, followed by Tukey’s post hoc test, with significance set at * *p* < 0.05 vs. grapes and ^†^ *p* < 0.05 vs. grape pomace. Abbreviations: M, myricetin; Q, quercetin; L, laricitrin; I, isorhamnetin; S, syringetin; K, kaempferol; glcU, glucuronide; glc, glucoside; gal, galactoside; dihex, dihexoside; hex, hexoside; rhm, rhamnoside; rut, rutinoside. ND, not detected. ^a^, negative ionization mode for phenolic compounds. ^b^, as quercetin-3-glucoside (Q-3-glc) equivalents. ^c^, as caftaric acid equivalents.

## Data Availability

The original contributions presented in the study are included in the article/Supplementary Material, further inquiries can be directed to the corresponding author.

## Supplementary Materials

The following supporting information can be downloaded at: https://www.mdpi.com/article/10.3390/nu16142312/s1, Table S1: Balanced chow composition (Nuvilab CR1®, Quimtia).

## References

[1] Rockenbach I.I., Gonzaga L.V., Rizelio V.M., de Souza Schmidt Gonçalves A.E., Genovese M.I., Genovese M.I., Fett R. (2011). Phenolic compounds and antioxidant activity of seed and skin extracts of red grape (*Vitis vinifera* and *Vitis labrusca*) pomace from Brazilian winemaking. Food Res. Int..

[2] Sousa E.C., Uchôa-Thomaz A.M.A., Carioca J.O.B., de Morais S.M., de Lima A., Travassos Ferreira P.A., Moreira Rodrigues A.L., Praciano Rodrigues S., do Nascimento Silva J., Lages Rodrigues L. (2014). Chemical composition and bioactive compounds of grape pomace (*Vitis vinifera* L.), Benitaka variety, grown in the semiarid region of Northeast Brazil. Food Sci. Technol..

[3] Zuin V.G., Ramin L.Z. (2018). Green and Sustainable Separation of Natural Products from Agro-Industrial Waste: Challenges, Potentialities, and Perspectives on Emerging Approaches. Top. Curr. Chem..

[4] Barcia M.T., Pertuzatti P.B., Rodrigues D., Bochi V.C., Hermosín-Gutiérrez I., Teixeira Godoy H. (2015). Effect of drying methods on the phenolic content and antioxidant capacity of Brazilian winemaking byproducts and their stability over storage. Int. J. Food Sci. Nutr..

[5] Jara-Palacios M.J., Hernanz D., Cifuentes-Gomez T., Escudero-Gilete M.L., Heredia F.J., Spencer J.P.E. (2015). Assessment of white grape pomace from winemaking as source of bioactive compounds, and its antiproliferative activity. Food Chem..

[6] Di Pietro Fernandes C., Santana L.F., Dos Santos J.R., Fernandes D.S., Hiane P.A., Pott A., de Cassia Freitas K., Bogo D., Aragao do Nascimento V., de Oliveira Filiu W.F. (2023). Nutraceutical Potential of Grape (*Vitis vinifera* L.) Seed Oil in Oxidative Stress, Inflammation, Obesity and Metabolic Alterations. Molecules.

[7] Bocsan I.C., Măgureanu D.C., Pop R.M., Levai A.M., Macovei Ș.O., Patrasca I.M., Chedea V.S., Buzoianu A.D. (2022). Antioxidant and Anti-Inflammatory Actions of Polyphenols from Red and White Grape Pomace in Ischemic Heart Diseases. Biomedicines.

[8] Schön C., Allegrini P., Engelhart-Jentzsch K., Riva A., Petrangolini G. (2021). Grape Seed Extract Positively Modulates Blood Pressure and Perceived Stress: A Randomized, Double-Blind, Placebo-Controlled Study in Healthy Volunteers. Nutrients.

[9] NCD Risk Factor Collaboration (NCD-RisC) (2021). Worldwide trends in hypertension prevalence and progress in treatment and control from 1990 to 2019: A pooled analysis of 1,201 population-representative studies with 104 million participants. Lancet.

[10] Acelajado M.C., Hughes Z.H., Oparil S., Calhoun D.A. (2019). Treatment of Resistant and Refractory Hypertension. Circ. Res..

[11] Teng H., Chen L. (2019). Polyphenols and bioavailability: An update. Crit. Rev. Food Sci. Nutr..

[12] Vázquez-Ruiz Z., Toledo E., Vitelli-Storelli F., Goni L., de la O.V., Bes-Rastrollo M., Martinez-Gonzalez M.A. (2022). Effect of Dietary Phenolic Compounds on Incidence of Cardiovascular Disease in the SUN Project; 10 Years of Follow-Up. Antioxidants.

[13] Singleton V.L., Orthofer R., Lamuela-Raventos R.M. (1999). Analysis of total phenols and others oxidation substrates and antioxidants by means of Folin-Ciocalteau Reagent. Methods Enzymol..

[14] Giusti M., Wrolstad R.E. (2001). Characterization and measurement of Anthocyanins by UV-Visible Spectroscopy. Curr. Protoc. Food Anal. Chem.

[15] Sarneckis C.J., Dambergs R.G., Jones P., Mercurio M.J. (2006). Herderich, P.A. et al. Quantification of condensed tannins by precipitation with methylcellulose: Development and validation of an optimised tool for grape and wine analysis. Aust. J. Grape Wine Res..

[16] Meléndez-Martínez A.J., Mandić A.I., Bantis F., Böhm V., Borge G.I.A., Brncic M., Bysted A., Cano M.P., Graca Dias M., Elgersma A. (2022). A comprehensive review on carotenoids in foods and feeds: Status quo, applications, patents, and research needs. Crit. Rev. Food Sci. Nutr..

[17] Castillo-Muñoz N., Gómez-Alonso S., García-Romero E., Hermosín-Gutiérrez I. (2007). Flavonol profiles of Vitis vinifera red grapes and their single-cultivar wines. J. Agric. Food Chem..

[18] Beres C., Costa G.N.S., Cabezudo I., da Silva-James N.K., Teles A.S.C., Cruz A.P.G., Mellinger-Silva C., Tonon R.V., Lourdes M.C.C., Freitas S.P. (2017). Towards integral utilization of grape pomace from winemaking process: A review. Waste Manag..

[19] Galanakis C.M. (2017). Handbook of Grape Processing By-Products-Sustainable Solutions.

[20] Unusan N. (2020). Proanthocyanidins in grape seeds: An updated review of their health benefits and potential uses in the food industry. J. Funct. Foods.

[21] Kammerer D., Claus A., Carle R., Schieber A. (2004). Screening of pomace polyphenols from red and white grape varieties (*Vitis vinifera* L.) by HPLC–DAD–MS/MS. J. Agric. Food Chem.

[22] Kammerer D., Claus A., Schieber A., Reinhold C. (2005). A new process for the recovery of polyphenols from grape pomace (*Vitis vinifera* L.). J. Food Sci..

[23] Rebello L.P.G., Lago-Vanzela E.S., Barcia M.T., Ramos A.M., Stringheta P.C., Da Silva R., Castillo-Munez N., Gomez-Alonso S., Hermosin-Gutierrez I. (2013). Phenolic composition of the berry parts of hybrid grape cultivar BRS Violeta (BRS Rubea×IAC 1398-21) using HPLC–DAD–ESI-MS/MS. Food Res. Int..

[24] Barcia M.T., Pertuzatti P.B., Gómez-Alonso S., Godoy H.T., Hermosín-Gutiérrez I. (2014). Phenolic composition of grape and winemaking by-products of Brazilian hybrid cultivars BRS Violeta and BRS Lorena. Food Chem..

[25] Farhadi K., Esmaeilzadeh F., Hatami M., Forough M., Molaie R. (2016). Determination of phenolic compounds content and antioxidant activity in skin, pulp, seed, cane and leaf of five native grape cultivars in West Azerbaijan province, Iran. Food Chem..

[26] Ferreira-Lima N.E., Burin V.M., Caliari V., Bordignon-Luiz M.T. (2016). Impact of Pressing Conditions on the Phenolic Composition, Radical Scavenging Activity and Glutathione Content of Brazilian Vitis vinifera White Wines and Evolution During Bottle Ageing. Food Bioprocess. Technol..

[27] Pertuzatti P.B., Mendonça S.C., Alcoléa M., Guedes C.T., Amorim F.E., Simoes Beckmann A.P., Almeida Gama L., Americo M.F. (2020). Bordo grape marc (*Vitis labrusca*): Evaluation of bioactive compounds in vitro and in vivo. LWT.

[28] Castillo-Muñoz N., Fernández-González M., Gómez-Alonso S., García-Romero E., Hermosín-Gutiérrez I. (2009). Red-color related phenolic composition of Garnacha Tintorera (*Vitis vinifera L.)* grapes and red wines. J. Agric. Food Chem..

[29] Ribéreau-Gayon P., Dubourdieu D., Donèche B., Lonvaud A. (2006). Handbook of Enology: The Microbiology of Wine and Vinifications.

[30] Ribéreau-Gayon P., Glories Y., Maujean A., Dubourdieu D. (2006). Handbook of Enology: The Chemistry of Wine Stabilization and Treatments.

[31] Colombo R.C., Roberto S.R., Nixdorf S.L., Pérez-Navarro J., Gómez-Alonso S., Mena-Morales A., Garcia-Romero E., Simoes Azeredo Goncalves L., Aparecida da Cruz M., de Carvalho D.U. (2020). Analysis of the phenolic composition and yield of ‘BRS Vitoria’ seedless table grape under different bunch densities using HPLC-DAD-ESI-MS/MS. Food Res. Int..

[32] Downey M.O., Dokoozlian N.K., Krstic M.P. (2006). Cultural Practice and Environmental Impacts on the Flavonoid Composition of Grapes and Wine: A Review of Recent Research. Am. J. Enol. Vitic..

[33] Ferrandino A., Carra A., Rolle L., Schneider A., Schubert A. (2012). Profiling of hydroxycinnamoyl tartrates and acylated anthocyanins in the skin of 34 *Vitis vinifera* genotypes. J. Agric. Food Chem..

[34] Pérez-Navarro J., Cazals. G., Enjalbal. C., Izquierdo-Cañas P.M., Gómez-Alonso S., Saucier C. (2019). Flavonol Glycoside Content of Grape Seeds and Skins of Vitis vinifera Varieties Grown in Castilla-La Mancha, Spain. Molecules.

[35] Simões C.M.O., Schenkel E.P., Mello J.C.P., Mentz L.A., Petrovick P.R. (2017). Pharmacognosy: From Natural Product to Medicine.

[36] Eskin N.A.M., Shahidi F. (2012). Food Biochemistry.

[37] Jackson D.I., Lombard P.B. (1993). Environmental and management practices affecting grape composition and wine quality-A review. Am. J. Enol. Vitic..

[38] Lewis K.V., Barros F., Cure M.B., Davies C.A., Furtado M.N., Hill T.C., Hirota M., Martins D.L., Mazzochini G.G., Mitchard E.T.A. (2022). Mapping native and non-native vegetation in the Brazilian Cerrado using freely available satellite products. Sci. Rep..

[39] Pellizzon M.A., Ricci M.R. (2018). The common use of improper control diets in diet-induced metabolic disease research confounds data interpretation: The fiber factor. Nutr. Metab..

[40] Del Pino-García R., Rivero-Pérez M.D., González-SanJosé M.L., Croft K.D., Muñiz P. (2017). Antihypertensive and antioxidant effects of supplementation with red wine pomace in spontaneously hypertensive rats. Food Funct..

[41] Jackson R.S. (2008). Wine Science–Principles and Applications.

[42] López-Fernández-Sobrino R., Soliz-Rueda J.R., Ávila-Román J., Arola-Arnal A., Suárez M., Muguerza B., Bravo F.I. (2021). Blood Pressure-Lowering Effect of Wine Lees Phenolic Compounds Is Mediated by Endothelial-Derived Factors: Role of Sirtuin 1. Antioxidants.

[43] Giles T.D., Sander G.E., Nossaman B.D., Kadowitz P.J. (2012). Impaired vasodilation in the pathogenesis of hypertension: Focus on nitric oxide, endothelial-derived hyperpolarizing factors, and prostaglandins. J. Clin. Hypertens..

[44] Goulopoulou S., Webb R.C. (2014). Symphony of vascular contraction: How smooth muscle cells lose harmony to signal increased vascular resistance in hypertension. Hypertension.

[45] Arzola-Rodríguez S.I., Muñoz-Castellanos L.N., López-Camarillo C., Salas E. (2022). Phenolipids, Amphipilic Phenolic Antioxidants with Modified Properties and Their Spectrum of Applications in Development: A Review. Biomolecules.

[46] Durazzo A., Lucarini M., Souto E.B., Cicala C., Caiazzo E., Izzo A.A., Novellino E., Santini A. (2019). Polyphenols: A concise overview on the chemistry, occurrence, and human health. Phytother. Res..

[47] Jara-Palacios M.J. (2019). Wine Lees as a Source of Antioxidant Compounds. Antioxidants.

[48] Alcaide-Hidalgo J.M., Martínez-Rodríguez A.J., Martín-Álvarez P.J., Pueyo E. (2008). Influence of the elaboration process on the peptide fraction with angiotensin I-converting enzyme inhibitor activity in sparkling wines and red wines aged on lees. Food Chem..

[49] Averilla J.N., Oh J., Kim H.J., Kim J.S., Kim J.S. (2019). Potential health benefits of phenolic compounds in grape processing by-products. Food Sci. Biotechnol..

[50] Schini-Kerth V.B., Auger C., Kim J.H., Etienne-Selloum N., Chataigneau T. (2010). Nutritional improvement of the endothelial control of vascular tone by polyphenols: Role of NO and EDHF. Pflug. Arch..

[51] Chacar S., Hajal J., Saliba Y., Bois P., Louka N., Maroun R.G., Faivre J.-F., Fares N. (2019). Long-term intake of phenolic compounds attenuates age-related cardiac remodeling. Aging Cell.

[52] Habauzit V., Morand C. (2012). Evidence for a protective effect of polyphenols-containing foods on cardiovascular health: An update for clinicians. Ther. Adv. Chronic Dis..

[53] Perdicaro D.J., Rodriguez Lanzi C., Fontana A.R., Antoniolli A., Piccoli P., Miatello R.M., Diez E.R., Vazquez Prieto M. (2017). Grape pomace reduced reperfusion arrhythmias in rats with a high-fat-fructose diet. Food Funct..

[54] Balea Ş.S., Pârvu A.E., Pop. N., Marín F.Z., Pârvu M. (2018). Polyphenolic Compounds, Antioxidant, and Cardioprotective Effects of Pomace Extracts from Fetească Neagră Cultivar. Oxid. Med. Cell. Longev..

[55] Ahmadi L., El-Kubbe A., Roney S.K. (2019). Potential Cardio-Protective Effects of Green Grape Juice: A Review. Curr. Nutr. Food Sci..

[56] Larson A.J., Symons J.D., Jalili T. (2012). Therapeutic potential of quercetin to decrease blood pressure: Review of efficacy and mechanisms. Adv. Nutr..

[57] Perez-Vizcaino F., Duarte J., Jimenez R., Santos-Buelga C., Osuna A. (2009). Antihypertensive effects of the flavonoid quercetin. Pharmacol. Rep..

[58] Popiolek-Kalisz J., Blaszczak P., Fornal E. (2022). Dietary Isorhamnetin Intake Is Associated with Lower Blood Pressure in Coronary Artery Disease Patients. Nutrients.

[59] Lucas Filho M.D. (2009). Phytochemical Study of Species of the Genus Erythroxylum with Potential Vasodilatory and Angiotensin-Converting Enzyme Inhibitory Activity. Master’s Thesis.

[60] Quaresma D.M.O. (2023). Phytochemical and Pharmacological Study of *Solanum subumbellatum* Vell. Ph.D. Thesis.

