# Supplementary material for: Enhancing Vascular Health and Lowering Blood Pressure in Spontaneously Hypertensive Rats through Syrah Grape (Vitis vinifera) Pomace: The Role of Phenolic Compounds
Source: Nutrients. 2024 Jul 18;16(14):2312. doi: 10.3390/nu16142312 (PMC11279649; doi:10.3390/nu16142312)
Supplement: Supplementary file 1 [file nutrients-16-02312-s001.zip › nutrients-3005296-supplementary.pdf]

SUPPLEMENTAL FILE

Table S1 - Balanced chow composition (Nuvilab CR1®, Quimtia)

Values per 100 g solid food item				
Total kcal	Total carbohydrate (g)	Protein (g)	Total fat (g)	Fiber (g)
350.5	55	22.5	4.5	8

Balanced chow composition: calcium carbonate, corn bran, soybean meal, wheat bran, dicalcium phosphate, sodium chloride, vitamin/mineral premix, amino acids, and antioxidant. Carbo hydrates: 53 to 55%; crude protein: 22 to 22.5%; lipids: 4.4 to 4.6%.

Composition: Ground whole corn, soybean bran, wheat bran, calcium carbonate, dicalcium phosphate, sodium chloride, vegetable oil, vitamin A, vitamin D3, vitamin E, vitamin K3, vitamin B1, vitamin B2, vitamin B6, vitamin B12, niacin, calcium pantothenate, folic acid, biotin, choline chloride, iron sulfate, manganese monoxide, zinc oxide, copper sulfate, calcium iodate, sodium selenite, cobalt sulfate, lysine, methionine, BHT . Guarantee levels per kilogram of product: Moisture (max.) 125 g/kg - Crude Protein (min.) 220 g/kg - Ethereal Extract (min.) 40 g/kg - Mineral Material (max.) 90 g/kg - Fibrous Matter (max.) 70 g/kg - Calcium (min-max.) 10 to 14 g/kg - Phosphorus (min.) 8,000 mg/kg. Supplementation per kilo of no less than: vitamins: Vitamin A (min) 13,000 IU/kg; vitamin D3 (min) 2,000 IU/kg; vitamin E (min) 34 IU/kg; vitamin K3 (min) 3 mg/kg; vitamin B1 (min) 5 mg/kg; vitamin B2 (min) 6 mg/kg; vitamin B6 (min) 7 mg/kg; vitamin B12 (min) 22 mcg/kg; niacin (min) 60 mg/kg; pantothenic acid (min) 20 mg/kg; folic acid (min) 1 mg/kg; biotin (min) 0.05 mg/kg; choline (min) 1,900 mg/kg. Mineral microelements: sodium (min) 2,700 mg/kg; iron (min) 50 mg/kg; manganese (min) 60 mg/kg; zinc (min) 60 mg/kg; copper (min) 10 mg/kg; iodine (min) 2 mg/kg; selenium (min) 0.05 mg/kg, cobalt (min) 1.5 mg/kg; fluorine (max) 80 mg/kg. Amino acids: Lysine (min) 12 g/kg; methionine (min) 4,000 mg/kg. Additives: BHT 100 mg/kg. Pellet measuring 15 to 16 mm in diameter, 30 to 40 mm in length (max). Compacted to avoid fragmentation.
